# Evaluation of four ionic liquids for pretreatment of lignocellulosic biomass

**DOI:** 10.1186/1472-6750-14-34

**Published:** 2014-04-30

**Authors:** John Gräsvik, Sandra Winestrand, Monica Normark, Leif J Jönsson, Jyri-Pekka Mikkola

**Affiliations:** 1 Department of Chemistry, Umeå University, Umeå SE-901 87, Sweden; 2 Industrial Chemistry & Reaction Engineering, Åbo Akademi University, Åbo-Turku FI-20500, Finland

**Keywords:** Ionic liquid, Pretreatment, Lignocellulose, Enzymatic saccharification

## Abstract

**Background:**

Lignocellulosic biomass is highly recalcitrant and various pretreatment techniques are needed to facilitate its effective enzymatic hydrolysis to produce sugars for further conversion to bio-based chemicals. Ionic liquids (ILs) are of interest in pretreatment because of their potential to dissolve lignocellulosic materials including crystalline cellulose.

**Results:**

Four imidazolium-based ionic liquids (ILs) ([C=C_2_C_1_im][MeCO_2_], [C_4_C_1_im][MeCO_2_], [C_4_C_1_im][Cl], and [C_4_C_1_im][HSO_4_]) well known for their capability to dissolve lignocellulosic species were synthesized and then used for pretreatment of substrates prior to enzymatic hydrolysis. In order to achieve a broad evaluation, seven cellulosic, hemicellulosic and lignocellulosic substrates, crystalline as well as amorphous, were selected. The lignocellulosic substrates included hybrid aspen and Norway spruce. The monosaccharides in the enzymatic hydrolysate were determined using high-performance anion-exchange chromatography. The best results, as judged by the saccharification efficiency, were achieved with [C_4_C_1_im][Cl] for cellulosic substrates and with the acetate-based ILs for hybrid aspen and Norway spruce. After pretreatment with acetate-based ILs, the conversion to glucose of glucan in recalcitrant softwood lignocellulose reached similar levels as obtained with pure crystalline and amorphous cellulosic substrates. IL pretreatment of lignocellulose resulted in sugar yields comparable with that obtained with acidic pretreatment. Heterogeneous dissolution with [C_4_C_1_im][HSO_4_] gave promising results with aspen, the less recalcitrant of the two types of lignocellulose included in the investigation.

**Conclusions:**

The ability of ILs to dissolve lignocellulosic biomass under gentle conditions and with little or no by-product formation contributes to making them highly interesting alternatives for pretreatment in processes where high product yields are of critical importance.

## Background

As petroleum and other fossil resources are not sustainable, renewable alternatives are needed for production of fuels, chemicals, and materials. Lignocellulosic biomass is an abundant non-food renewable resource that can contribute to a shift towards a sustainable bio-based society [1]. The three major constituents of lignocellulosic biomass are cellulose, hemicelluloses, and lignin. Cellulose and hemicelluloses are polysaccharides that can be hydrolyzed to monosaccharide sugars. The monosaccharides obtained can then be utilized for production of ethanol or other commodities [2].

Saccharification is typically accomplished through enzymatic hydrolysis, as the high selectivity of enzymatic catalysis gives high sugar yields. A pretreatment step is needed to achieve efficient bioconversion of lignocellulosic feedstocks, as the cellulose fraction is not easily accessible to enzymes [3,4]. However, many pretreatment methods require harsh conditions and give rise to by-products, which decrease the sugar yields and inhibit enzymatic and microbial biocatalysts.

Solvents that dissolve cellulose are of interest for pretreatment of lignocellulosic feedstocks prior to bioconversion. In nature, cellulose occurs mainly as highly crystalline cellulose I [5]. The crystallinity may prevent efficient enzymatic conversion and it is therefore desirable to disrupt the structure of the cellulose. Since cellulose is amphiphilic [6,7], it has been suggested that both hydrophobic and hydrophilic interactions need to be broken to dissolve cellulose. Therefore, a suitable solvent system should contain a co-solvent that weakens the hydrophobic interactions in an aquatic environment [8]. Several solvent systems for cellulose are known [9-14], but many of them suffer from problems like high toxicity and being difficult to recycle. Ionic liquids [ILs] are non-volatile solvents that can dissolve cellulose under gentle conditions.

Several attempts have been made to utilize ILs for pretreatment of cellulose [15-19]. Aspects that have been studied include the inhibitory effect on enzymes [20-22], the influence of water content [23,24] and cellulose load [25], the particle size of the biomass [26-28], the pretreatment temperature [29], and the pretreatment time [18]. However, an extensive study on the pretreatment effects of different ILs on different types of cellulosic substrates is lacking. Comparisons have been made, but they are based on biomass from different sources and treatments performed under different conditions [30]. Thus, the aim of this study was to investigate the pretreatment effect of different ILs on different types of substrates under identical conditions. Four ILs (Table 1) and seven substrates (Table 2) were studied.

**Table 1:** Results from the ionic liquid synthesis

**Abbreviation**	**Structure**	**Name**	**H**_**2**_**O cont.**	**Cl**^**-**^**cont.**	**Yield**^**a**^**(%)**
[C_4_C_1_im][MeCO_2_]		1-butyl-3-methylimidazolium acetate	260 ppm	0.8%	37
[C_4_C_1_im][HSO_4_]		1-butyl-3-methylimidazolium hydrogen sulfate	20%	N.A.^b^	87
[C_4_C_1_im][Cl]		1-butyl-3-methylimidazolium chloride	940 ppm	N.A.	48
[C=C_2_C_1_im][MeCO_2_]		1-allyl-3-methylimidazolium acetate	1500 ppm	0.9%	36

^a^The yields were calculated for the total synthesis (two steps for all of the ILs except [C_4_C_1_im][Cl], which was synthesized in one step) starting from the substituted imidazoles. ^b^Not Applicable.

**Table 2:** Cellulosic, hemicellulosic and lignocellulosic substrates used in the investigation

**Category**	**Substrate**	**Description**	**Source/reference**
Crystalline samples	Cotton	Linear cellulosic homopolysaccharide of β-D-glucopyranosyl units (95-99%), crystallinity: 64.9%, typical DP: 8,100 - 15,300.	Selefatrade AB/[31-33]
	Sigmacell Type 20	Linear cellulosic homopolysaccharide of β-D-glucopyranosyl units, crystallinity: 36.1%, DP: 209.	Sigma-Aldrich/[34,35]
Amorphous samples	Regenerated amorphous cellulose	Amorphous cellulosic homopolysaccharide prepared from Sigmacell Type 20 using phosphoric acid.	This work/Prepared using method of Zhang *et al.*[36].
	Beech-wood xylan	Branched hemicellulosic heteropolysaccharide, typical DP of hardwood xylan: 100–200.	Sigma-Aldrich/[32]
	Locust bean gum galactomannan	Branched hemicellulosic heteropolysaccharide, DP: 900–1500.	Sigma-Aldrich/[32]
Wood samples	Hybrid aspen (*Populus tremula* x *tremuloides*)	Hardwood lignocellulose, see Table 3.	Umeå Plant Science Centre (<http://www.upsc.se>)/this work
	Norway spruce (*Picea abies*)	Softwood lignocellulose, see Table 3.	SEKAB AB/this work

The four ILs (Table 1) were chosen since they have been used to dissolve cellulosic substrates and are recognized for their performance in such applications. Also, the set studied would allow for the detection of performance variations between different types of ILs used for this purpose. [C_4_C_1_im][Cl] is one of the most studied ILs when it comes to dissolving cellulose and serves as a good reference [15,17,30,37]. [C_4_C_1_im][HSO_4_] is highly interesting because it has been reported to dissolve lignin in high water content [10-40% (w/w) water], which is substantially more than [Cl]^-^- and [MeCO_2_]^-^-based ILs can handle [18]. [C_4_C_1_im][MeCO_2_] has the highest basicity among the selected [C_4_C_1_im]-based ILs, a property that is strongly linked to the capability of the IL to dissolve cellulose [30,37]. Finally, [C=C_2_C_1_im][MeCO_2_] was selected because of its high reactivity, low viscosity, and ability to dissolve cellulose [25]. It has been suggested that π-π interactions between the allyl side chain and lignin will improve lignin solubility [38]. The set of ILs chosen also gives the possibility to investigate the effect of the cation, by comparing the results from [C=C_2_C_1_im][MeCO_2_] with those from [C_4_C_1_im][MeCO_2_]. With regard to dissolution of wood saw dust and thermo-mechanical wood pulp, the performance of 1-allyl-3-methylimidazolium chloride [C=C_2_C_1_im][Cl] even surpassed that of [C_4_C_1_im][Cl] [38]. The set of ILs studied (Table 1) would also permit a comparison of ILs that are capable of homogeneous dissolution of lignocellulose, i.e. complete dissolution of all the main components of lignocellulose, and ILs known to be restricted to heterogeneous dissolution of lignocellulose [30]. [C_4_C_1_im][MeCO_2_], [C_4_C_1_im][Cl] and [C=C_2_C_1_im][MeCO_2_] should give rise to homogeneous dissolution of lignocellulose (i.e. dissolve cellulose, hemicellulose and lignin). [C_4_C_1_im][HSO_4_] would effect heterogeneous dissolution of lignocellulose by selectively dissolving lignin and hemicellulose, but would not dissolve cellulose.

The study included cellulosic (cotton, Sigmacell Type 20, and regenerated amorphous cellulose), hemicellulosic (xylan and galactomannan), as well as lignocellulosic (hybrid aspen and Norway spruce) substrates (Table 2). The selected collection of substrates differ widely with regard to fundamental properties, such as crystallinity, degree of polymerization (DP), and complexity (Table 2). The most complex substrates, aspen and spruce, represent hardwood and softwood, respectively. Softwood is typically the most recalcitrant type of lignocellulosic biomass [4].

The sets of ILs and substrates used in the investigation would allow a broad evaluation of the ILs with regard to the effects of the anion and the cation as well as the importance of homogeneous and heterogeneous dissolution on the pretreatment efficiency. To permit a comparison of IL pretreatment with a conventional state-of-the-art pretreatment method, one of the lignocellulosic samples was also pretreated using classical sulfuric acid pretreatment. Using in-house prepared ILs and advanced analytical methods such as high-performance anion-exchange chromatography (HPAEC) to quantify different monosaccharides released from the polymeric substrates by the hydrolytic enzymes, this investigation addresses the lack of direct comparisons of the effects of ILs on cellulosic, hemicellulosic, and lignocellulosic samples.

## Results and discussion

### Ionic liquids

**Figure 1:**
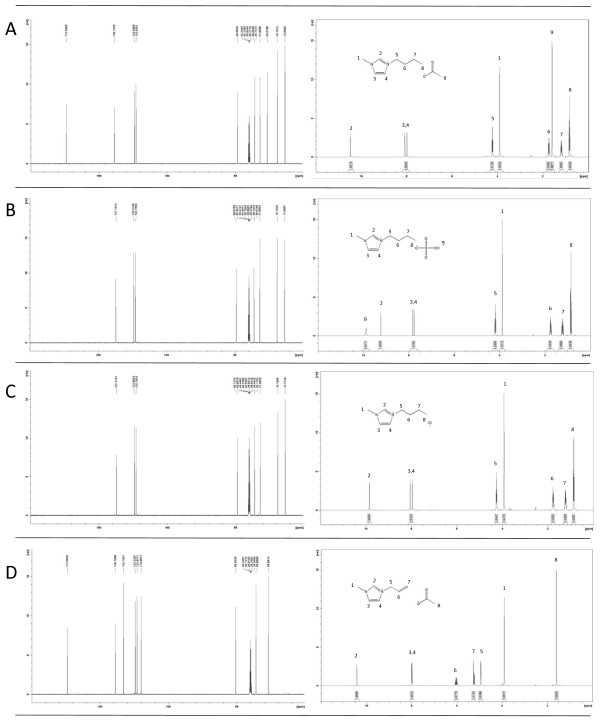
**NMR analysis of ionic liqid preparations: (A) 1-butyl-3-methylimidazolium acetate, (B) 1-butyl-3-methylimidazolium hydrogen sulfate, (C) 1-butyl-3-methylimidazolium chloride, and (D) 1-allyl-3-methylimidazolium acetate.** Data of ^13^C NMR (left side) and ^1^H NMR (right side) analysis are shown.

Data on the properties and synthesis of the four ionic liquids synthesized as a part of the investigation are shown in Table 1. Figure 1 shows the results of ^1^H and ^13^C NMR analysis of [C_4_C_1_im][MeCO_2_], [C_4_C_1_im][HSO_4_], [C_4_C_1_im][Cl], and [C=C_2_C_1_im][MeCO_2_]. The figure shows the peak assignments for the ^1^H NMR spectra. The clean spectra indicate that the ionic liquids were of high purity. Dialkylated imidazoles, such as [C_4_C_1_im][MeCO_2_], [C_4_C_1_im][HSO_4_] and [C_4_C_1_im][Cl] (Table 1), are among the more common cations used for the dissolution of biomass. In fact, 1-butyl-3-methylimidazolium chloride ([C_4_C_1_im][Cl]) was one of the first ILs used to dissolve cellulose [15] and lignocellulose [27]. The focus has recently shifted from chloride-based to acetate-based ILs [26], which in this study are represented by [C_4_C_1_im][MeCO_2_] and [C=C_2_C_1_im][MeCO_2_] (Table 1). However, the stability of acetate-based ILs is not as good as that of chloride-based ILs, such as [C_4_C_1_im][Cl] (Table 1).

The Kamlet-Taft polarity, or, more precisely, the hydrogen-bond basicity of the IL, is believed to be strongly linked to its capability to dissolve or to swell lignocellulose [23,39]. However, the basicity of an IL is related to its hydrophilicity [40] and poses a problem which has to do with the water content of the IL. In equilibrium with air, ILs with acetate as anion can reach a water content of 27% (w/w) [41]. However, even a relatively low content of water (3-5% (w/w) in a study of *Miscanthus*[28] and 5-10% (w/w) in a study of maple-wood flour [24]) has been shown to have a negative effect on the solubility of lignocellulose. This is not optimal in a process, where the energy used for drying the biomass and the IL needs to be taken in to account.

The water content of the ILs (Table 1) was determent by Karl-Fischer titration after the samples had been exposed to high vacuum (<50 mbar) at 65°C overnight. The water content of all ILs was below 0.16 wt%, except for [C_4_C_1_im][HSO_4_], which on purpose had a high water content. According to previous studies [18], the sugar yield from lignocellulose pretreated with [C_4_C_1_im][HSO_4_] was significantly higher when the water content was 10-40%. The water content of the [C_4_C_1_im][HSO_4_] used in our study, 20% (Table 1), is thus within the desired range.

The yields of the ILs (Table 1) were calculated for the total synthesis starting from the corresponding 1-alkyl-imidazole (methyl and butyl). This explains why the yields were sometimes relatively low (Table 1). [C_4_C_1_im][MeCO_2_] and [C=C_2_C_1_im][MeCO_2_] were made from the corresponding saturated and unsaturated 1-alkyl-3-methylimidazolium chlorides. The ion exchange was tracked using NMR to ensure that the acetate ion was duly present and that the ion exchange was successful. The halide contamination was controlled by using HPAEC and a sodium chloride standard. The chloride contamination was <1% (Table 1).

### Biomass samples

The amounts of structural carbohydrates and lignin were determined after extraction of the aspen and the spruce wood (Table 3). The values correspond well with those found in the literature [42]. The glucan content of the aspen and spruce wood was rather similar, around 40% (Table 3). As expected, the aspen wood had a relatively high xylan content (16.6%), while the spruce wood had relatively high mannan content (10.4%). The total lignin content of Norway spruce (28.3%) was higher than that of hybrid aspen (23.4%). The lignin content is highly relevant for enzymatic hydrolysis of cellulose [4], as lignin can obstruct saccharification by forming a physical barrier, by catalytically unproductive binding of hydrolytic enzymes, and by formation of lignin-carbohydrate complexes (LCCs).

**Table 3:** **Contents of structural carbohydrates and lignin in aspen and spruce**^**a**^

**Biomass**	**Glucan**	**Arabinan**	**Galactan**	**Xylan**	**Mannan**	**Total lignin**^**b**^
Hybrid aspen	0.409 ± 0.015	0.008 ± 0.001	0.016 ± 0.001	0.166 ± 0.010	0.030 ± 0.012	0.234 ± 0.002
Norway spruce	0.394 ± 0.003	0.014 ± 0.001	0.020 ± 0.001	0.053 ± 0.001	0.104 ± 0.001	0.283 ± 0.002

^a^Reported as g of carbohydrate or lignin per g of wood (dry weight) with standard deviations.

^b^Klason lignin and acid-soluble lignin.

### Pretreatment and enzymatic saccharification

Cellulosic, hemicellulosic and lignocellulosic samples (5% wt) were pretreated with the four ILs at 100°C for 20 h. The precipitation was carried out using a small amount of methanol and ultra-pure deionized water. The precipitate was collected by centrifugation and washed three times with ultra-pure water and once with citrate buffer prior to the enzymatic saccharification. The enzyme mixture used contains a wide variety of proteins, which are expected to degrade not only cellulose, but also xylan and mannan. The use of a sensitive analytical method for determination of the yields of the monosaccharides (HPAEC) would allow detection of small quantities of sugars such as xylose and mannose. The enzymatic saccharification was carried out analytically in a small scale (reactions with 50 mg sample), which made the processing of multiple parallel samples and the evaluation of the experiments using statistical analysis feasible.

#### Crystalline samples

**Figure 2:**
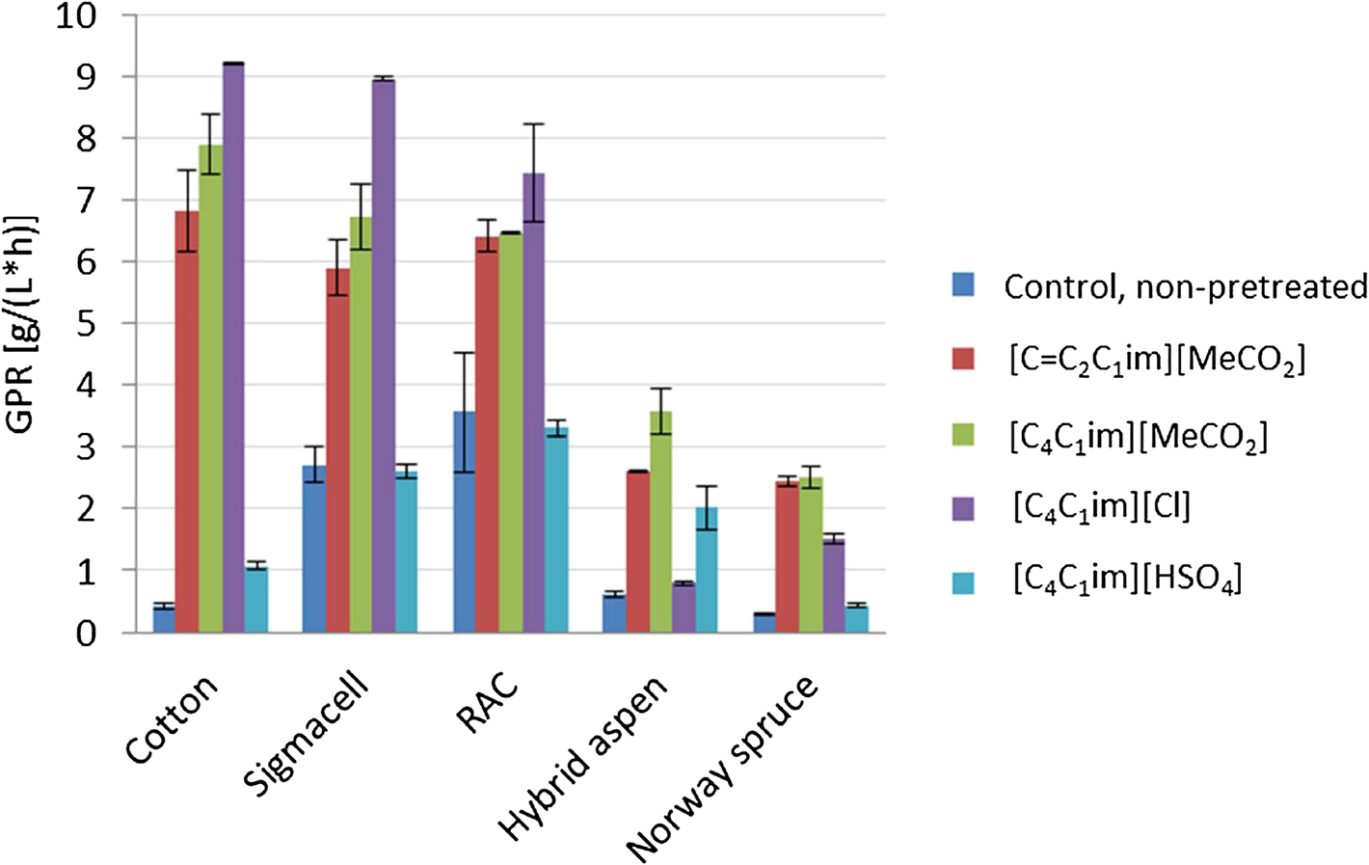
**Glucose production rate (GPR) in g glucose l**^**-1**^ **h**^**-1**^**after 2 h of enzymatic hydrolysis (before the reaction rates level off) for the ionic-liquid-pretreated samples and the non-pretreated control.**

**Figure 3:**
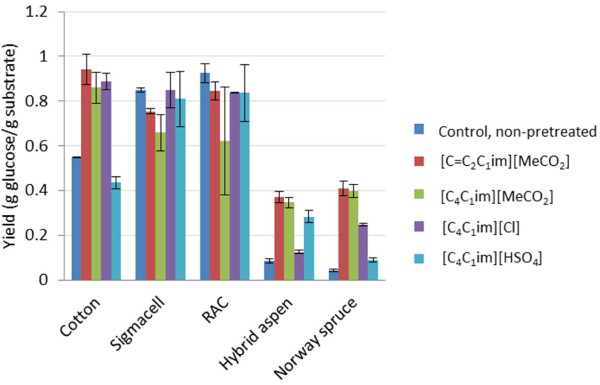
**The glucose yield in g glucose g substrate**^**-1**^**after 72 h of enzymatic hydrolysis (at the end of the reactions after that the reaction rates level off) of ionic-liquid-pretreated samples and non-pretreated control.**

Compared to the untreated control, the glucose production rate [GPR, value based on the glucose concentration after 2 h before the reaction levels off (see Section Glucose concentration estimation)] of cotton increased dramatically after treatment with [C_4_C_1_im][Cl], [C_4_C_1_im][MeCO_2_], and [C=C_2_C_1_im][MeCO_2_] (Figure 2). For cellulosic samples, pretreatment with [C_4_C_1_im][Cl] gave the best results (Figure 2). The GPR increased significantly (P ≤ 5%, t-test) also for the [C_4_C_1_im][HSO_4_]-pretreated sample compared to that of the control sample. Compared to the untreated control, the glucose yield [value based on the glucose concentration after 72 h, when the reaction has leveled off (see Section Analysis of monosaccharide yields)] increased significantly after pretreatment with all of the ILs except [C_4_C_1_im][HSO_4_] (Figure 3). Pretreatment of cotton with [C_4_C_1_im][MeCO_2_], [C=C_2_C_1_im][MeCO_2_] and [C_4_C_1_im][Cl] increased the yield with 160, 170 and 160%, respectively. The difference can be attributed to that [C_4_C_1_im][HSO_4_] causes heterogeneous dissolution of lignocellulose, while the other three ILs cause homogeneous dissolution. As cotton consists almost of pure cellulose (Table 2), the capacity of [C_4_C_1_im][HSO_4_] to dissolve lignin and hemicellulose would be of little or no help. For [C_4_C_1_im][MeCO_2_], [C=C_2_C_1_im][MeCO_2_] and [C_4_C_1_im][Cl], the increase in GPR (Figure 2) was higher than the increase in yield (Figure 3), which indicates that the effect of the pretreatment is most beneficial for the initial phase of the hydrolysis of the cellulose.

With regard to the microcrystalline Sigmacell Type 20 cellulose, the GPRs of samples pretreated with [C_4_C_1_im][Cl], [C_4_C_1_im][MeCO_2_] and [C=C_2_C_1_im][MeCO_2_] were significantly higher (P ≤ 5%, t-test) than that of the control (Figure 2). The glucose yields of pretreated and non-pretreated samples did not exhibit any significant differences (Figure 3). Treatment with [C_4_C_1_im][HSO_4_] did not result in any improvement for either the GPR or the glucose yield, as could be expected since [C_4_C_1_im][HSO_4_] does not dissolve cellulose.

When untreated Sigmacell Type 20 was used as substrate instead of untreated cotton, the GPR increased from 0.41 g l^-1^ h^-1^ to 2.72 g l^-1^ h^-1^ and the glucose yield increased from 0.55 g g^-1^ to 0.85 g g^-1^. Evidently, the Sigmacell Type 20 cellulose was more susceptible to enzymatic hydrolysis than cotton cellulose. The difference can probably be attributed to the much lower degree of polymerization (DP) of Sigmacell Type 20 compared to cotton (Table 2), which would give the exo-acting cellobiohydrolases a larger number of cellulose chain ends to attack. It is also possible that a lower degree of crystallinity of Sigmacell Type 20 cellulose (Table 2) contributes to facilitating the enzymatic hydrolysis.

After pretreatment of cotton with [C_4_C_1_im][Cl], [C_4_C_1_im][MeCO_2_] or [C=C_2_C_1_im][MeCO_2_], the GPR values were similar or slightly higher than the corresponding GPR values for Sigmacell Type 20 (Figure 2). This was not due to that the substrate had been exhausted already after 2 h, as less than 35% of the substrate had been converted to glucose. The yield values (Figure 3) from samples taken after 72 h were also lower than the theoretical maximum, which would be approx. 1.1 g glucose per g cellulose. The highest yield observed for the two cellulosic crystalline substrates, 0.94 g g^-1^, was obtained after pretreatment of cotton with [C=C_2_C_1_im][MeCO_2_] and corresponds to conversion of around 85% of the substrate to glucose. Thus, although the natural cotton was more recalcitrant than Sigmacell Type 20, pretreatment with all of the ionic liquids except [C_4_C_1_im][HSO_4_] removed that effect.

#### Amorphous samples

The GPR values (Figure 2) and the glucose yield values (Figure 3) for regenerated amorphous cellulose (RAC) were rather similar to the values of the Sigmacell Type 20 cellulose that the RAC was derived from. This suggests that the crystallinity of the Sigmacell Type 20 cellulose (Table 2) was not an obstacle for the enzymatic hydrolysis. Apart from conversion of crystalline cellulose to amorphous, treatment with acid may potentially also result in hydrolysis which could give decreased cellulose chain length, but evidently this did not lead to improved hydrolysis of RAC compared to Sigmacell Type 20 under the experimental conditions studied.

Enzymatic hydrolysis of untreated or IL-pretreated hemicellulosic samples for 72 h resulted in very low yields (<0.05 g g^-1^) of glucose and arabinose. Enzymatic hydrolysis of untreated beech-wood xylan gave a xylose yield of 0.55 ± 0.09 g g^-1^, while untreated locust bean gum galactomannan gave a galactose yield of 0.18 ± 0.01 g g^-1^ and a mannose yield of 0.54 ± 0.03 g g^-1^. After pretreatment of the beech-wood xylan with ILs, the xylose yields compared to that of the untreated control were 23% for [C=C_2_C_1_im][MeCO_2_], 25% for [C_4_C_1_im][MeCO_2_], 39% for [C_4_C_1_im][Cl], and 7% for [C_4_C_1_im][HSO_4_]. For the locust bean gum galactomannan, the galactose yields compared to that of the untreated control were 33% for [C=C_2_C_1_im][MeCO_2_], 75% for [C_4_C_1_im][MeCO_2_], and 66% for [C_4_C_1_im][Cl]. No galactose was recovered from galactomannan pretreated with [C_4_C_1_im][HSO_4_]. Finally, the mannose yields compared to that of the untreated galactomannan control were 91% for [C_4_C_1_im][MeCO_2_], 73% for [C_4_C_1_im][Cl], and 3% for [C_4_C_1_im][HSO_4_]. No mannose was recovered after pretreatment of galactomannan with [C=C_2_C_1_im][MeCO_2_]. Thus, the recovery of hemicellulose sugars after pretreatment with ILs was always poor.

A possible explanation for poor recovery of hemicellulosic sugars is that previous studies have shown that ILs with a high basicity will hydrolyze hemicellulose to oligomeric sugars [43]. It has also been shown that sulfate-based ILs, especially [HSO_4_]^-^, will hydrolyze hemicellulose to monomeric sugars and even dehydrate pentose sugars to furfural [18]. Poor recovery of hemicellulose sugars has also been indicated in previous studies [30].

#### Wood samples

As expected considering the recalcitrance of softwood, untreated Norway spruce exhibited lower GPR (0.28 ± 0.02 g l^-1^ h^-1^) and glucose yield (0.043 ± 0.006 g g^-1^) than untreated hybrid aspen (GPR, 0.62 ± 0.04 g l^-1^ h^-1^; glucose yield, 0.085 ± 0.01 g g^-1^). Pretreatment of the wood samples with the four ILs resulted in significantly higher (P ≤ 5%, t-test) GPR values and glucose yields (Figures 2 and 3). The increase of the yields (Figure 3) varied between 150 and 950% compared to that of the untreated wood samples.

The highest yields were obtained upon use of [C_4_C_1_im][MeCO_2_] and [C=C_2_C_1_im][MeCO_2_] (Figure 3). For aspen, the glucose yields with [C_4_C_1_im][MeCO_2_] and [C=C_2_C_1_im][MeCO_2_] were 0.35 ± 0.02 g g^-1^ and 0.37 ± 0.02 g g^-1^, respectively. These yields were slightly higher than that achieved in a control experiment with dilute sulfuric acid pretreatment and enzymatic hydrolysis of aspen, which resulted in a glucose yield of 0.31 ± 0.05 g g^-1^. For aspen, the glucan conversion after pretreatment with [C_4_C_1_im][MeCO_2_] and [C=C_2_C_1_im][MeCO_2_] was 76% and 81%, respectively. When spruce was pretreated using [C_4_C_1_im][MeCO_2_] or [C=C_2_C_1_im][MeCO_2_], more than 90% of the glucan was converted to glucose. That is a higher value than achieved with the pure cellulosic crystalline and amorphous substrates, which indicates the potential of using ILs for pretreatment of lignocellulose.

The glucose yields after pretreatment of aspen with the ILs that did not have acetate as anion were lower and corresponded to 0.28 ± 0.03 g g^-1^ for [C_4_C_1_im][HSO_4_] and 0.13 ± 0.01 g g^-1^ for [C_4_C_1_im][Cl]. The glucan conversions after pretreatment of aspen with [C_4_C_1_im][Cl] and [C_4_C_1_im][HSO_4_] were 28% and 62%, respectively. The corresponding values for spruce were 57% and 20%. While [C_4_C_1_im][HSO_4_] did not work very well for pretreatment of the recalcitrant spruce wood, the results obtained with aspen were not far behind the results achieved with acetate-based ILs (Figures 2 and 3).

There were no significant differences between [C_4_C_1_im][MeCO_2_] and [C=C_2_C_1_im][MeCO_2_] in terms of pretreatment of any of the samples in this study. This indicates that the choice of cation is often not decisive in terms of the efficiency of the pretreatment process. The higher yields obtained upon use of ionic liquids with an acetate anion can most likely be explained by the higher basicity of the acetate compared to the chloride and hydrogen sulfate (reviewed by Brandt *et al.*[30]). The comparatively low yields found for the hydrogen sulfate anion compared to the acetates can be attributed to the fact that it does not dissolve cellulose. However, it does dissolve lignin and hemicelluloses, which would explain the higher yields achieved compared to non-pretreated wood samples.

## Conclusions

Four ILs were evaluated for pretreatment of seven cellulosic, hemicellulosic and lignocellulosic substrates prior to enzymatic saccharification. The wide variety of substrates investigated and the use of the same pretreatment conditions for all ILs studied facilitated comparisons of the performance of the different ILs.

Although they were not the most efficient pretreatment agents for simple cellulosic substrates, ILs with acetate as anion gave the best results for complex lignocellulosic substrates from wood. However, among the ILs used in this study the ones with acetate as anion are among the least stable. Moreover, they are cumbersome to synthesize, and they are sensitive to moisture.

The halide-based IL [C_4_C_1_im][Cl] performed very well for simple cellulosic substrates, but was less efficient for pretreatment of wood. [C_4_C_1_im][Cl] possesses advantages from the process perspective, since it is easy to synthesize, relatively stable, and less sensitive to moisture than the acetate-based ILs. Disadvantages with [C_4_C_1_im][Cl] include its high melting point and high viscosity.

The difference between homogeneous and heterogeneous dissolution of lignocellulose was studied by the inclusion of [C_4_C_1_im][HSO_4_] in the set of ILs studied. Homogeneous dissolution was not always better, as [C_4_C_1_im][HSO_4_] gave better results than [C_4_C_1_im][Cl] with hybrid aspen, the less recalcitrant type of lignocellulose included in the study. This warrants further attention in future studies, for example by optimization of the pretreatment process.

The best choices, as judged by the saccharification efficiency, appeared to be [C_4_C_1_im][Cl] for cellulosic substrates, acetate-based ILs or [C_4_C_1_im][HSO_4_] for hybrid aspen, and acetate-based ILs for Norway spruce. Pretreatment can, however, involve high water content, something that has always been a problem with homogeneous dissolution of lignocellulose. The possibility to include water and the stability of [C_4_C_1_im][HSO_4_] have potential to decrease the cost for the regeneration process. Therefore other factors than the capacity to dissolve cellulose, such as IL stability, the possibility to include water, and regeneration of the IL, need to be considered when choosing ILs that are most suited for industrial pretreatment.

Conversion of crystalline Sigmacell Type 20 cellulose to RAC did not improve enzymatic hydrolysis. This was shown for both non-pretreated cellulose and cellulose that was pretreated with different ILs. ILs that enhanced hydrolysis of the crystalline form also enhanced hydrolysis of the RAC.

Pretreatment with ILs gave sugar yields comparable with those obtained with acidic pretreatment technique. Pretreatment with ILs can be performed using gentle conditions, while acidic pretreatment is typically performed at high temperature and high pressure, and in a corrosive environment, factors that increase the demands on the quality of the process equipment and contributes to lower yields due to the formation of by-products from the lignocellulose feed. High yields are critical in the production of biofuels and other bulk chemicals. ILs also have low vapor pressure which is advantageous with regard to safety and environmental aspects. Thus, provided that ILs used in industrial processes can be reused, there are distinct advantages that make further research on the use of ILs for conversion of lignocellulose worthwhile. Future areas of research include recycling of ILs, up-scaling, process integration, and techno-economical assessment.

## Methods

### Ionic liquid synthesis

#### Purification of starting materials

1-Methylimidazole (Sigma-Aldrich, 99%) and 1-butylimidazole (Aldrich, 99%) were distilled under vacuum from potassium hydroxide (Akzo Nobel, 87.9%). 1-Chlorobutane (Acros Organics, 99%), allyl chloride (Aldrich, 99%) and dimethylsulfate (Fluka, 95%) were distilled from CaO (Sigma-Aldrich). Ethyl acetate (Fisher Chemicals HPLC) and acetonitrile (Fisher Chemicals HPLC) were dried by running them through neutral aluminum oxide 90 (Merck 70–230 mesh ASTM) and toluene (Sigma-Aldrich, 99.7%) through silica (Acros Organics, Ultrapure 40–60 μm, 60A). All starting materials were used within hours of preparation. All reactions and washing procedures were carried out in dry glassware with dried chemicals and under inert atmosphere (argon) if not stated otherwise or in the case where water was used.

#### [C_4_C_1_im][Cl]

1-Methylimidazole (99 ml, 1.2 mol) was dissolved in 150 ml ethyl acetate and stirred at room temperature. 1-Chlorobutane (130 ml, 1.2 mol) was added dropwise to the reaction mixture and afterwards the reaction mixture was refluxed at 75°C for 4 days. The reaction vessel was allowed to cool at room temperature and was then thoroughly sealed and was kept at -20°C overnight. The upper organic layer was then removed using Schlenk techniques and the bottom IL layer was washed two times with 75 ml ethyl acetate. After the first wash, the IL precipitated into white crystals that were then dissolved in approximately 50 ml of acetonitrile and were recrystallized overnight at -20°C. The upper organic layer was again removed, and the crystals were washed three times with 50 ml of ethyl acetate and dried overnight using vacuum treatment in order to remove the last of the solvents. The yield was 48%.

#### [C=C_2_C_1_im][Cl]

Allyl chloride (116 ml, 1.43 mol) was added dropwise to 1-methylimidazole (92 ml, 1.14 mol) under stirring and cooled in an ice-bath. Afterwards the reaction mixture was refluxed for 14 h at which point the reflux condenser was replaced with a distillation head and the reaction mixture was vacuum-distilled to give an amber-colored viscous liquid. The yield was 96%.

#### [C_4_C_1_im][MeCO_2_]

[C_4_C_1_im][MeCO_2_] was prepared from [C_4_C_1_im][Cl] using an ion-exchange column. Amberlite IRA-400 (OH^-^ form) ion-exchange resin (64 g; enough to support the conversion of 20 g [C_4_C_1_im][Cl]) was dispersed in deionized water and poured into a glass column (3 cm internal diameter). The Amberlite column was then loaded with [MeCO_2_]^-^ by running through 1000 ml of a 1 M aqueous solution of [Na][MeCO_2_] using a flow rate of 0.8 ml/min. Once the correct ion was loaded on the column, 15.3 g of [C_4_C_1_im][Cl] was dissolved in 60 ml of deionized water and poured onto the Amberlite column with a flow-rate of 0.2 ml/min. When all the [C_4_C_1_im][Cl] aqueous solution had passed into the column, another 10 ml of water was added and were allowed to run through in the same flow-rate. Fractions were collected during this time, and analyses with HPAEC were used to determine the chloride content. All fractions were evaporated and pumped overnight at 65°C under stirring to remove the water eluent. The chloride content was below 0.8% (mol/mol). Afterwards, the Amberlite column was rejuvenated with 1000 ml of a 1 M aqueous solution of NaOH and with a flow-rate of 0.8 ml/min. The yield was 78%

#### [C=C_2_C_1_im][MeCO_2_]

[C=C_2_C_1_im][MeCO_2_] was prepared from [C=C_2_C_1_im][Cl] by using an ion-exchange column. A dispersion of Amberlite IRA-400 (OH^-^ form) ion-exchange resin (64 g; enough to support the conversion of 20 g [C_4_C_1_im][Cl]) was prepared in deionized water and poured into a glass column (3 cm internal diameter). The Amberlite column was then loaded with [MeCO_2_]^-^ by running through 1000 ml of a 1 M aqueous solution of [Na][MeCO_2_] with a flow-rate of 0.8 ml/min. Once the correct ion was loaded on the column, 15.2 g of [C=C_2_C_1_im][Cl] was dissolved in 100 ml of deionized water and poured on the Amberlite column and the flow-rate was set to 0.2 ml/min. When all the [C=C_2_C_1_im][Cl] aqueous solution had passed into the column, another 10 ml of water was added and were allowed to run through in the same flow-rate. Fractions were collected during this time and analyses with HPAEC were performed to determine the chloride content. All fractions were subjected to a drying process in evaporator and high vacuum overnight at 65°C under stirring to remove the water eluent. The chloride content was found to reside below 0.9% (mol/mol). Afterwards, the Amberlite column was rejuvenated with 1000 ml of a 1 M aqueous solution of sodium hydroxide with a flow-rate of 0.8 ml/min. The yield was 76%.

#### [C_4_C_1_im][MeSO_4_]

1-Butylimidazole (51 ml, 0.39 mol) was diluted in 60 ml toluene and cooled in an ice bath. Dimethyl sulfate (37 ml, 0.39 mol) was then added drop-wise (flow rate 0.5 drops/sec) and under stirring to the reaction mixture. Afterwards the reaction mixture was stirred for another hour at room temperature before the upper toluene layer was removed using Schlenk techniques. The lower IL layer was subsequently washed three times with 50 ml of toluene before it was dried overnight at 65°C under vacuum and stirring. The yield obtained was 89%.

#### [C_4_C_1_im][HSO_4_]

[C_4_C_1_im][MeSO_4_] (29.9 g) was diluted in 5 ml deionized water. The reaction vessel was kept open to the air and put in a heating block at 215°C. The internal temperature of the reaction mixture was continuously monitored and kept between 170 to 180°C for four hours by drop-wise addition of deionized water. Afterwards, the reaction mixture was cooled to 65°C and was kept under vacuum and stirring overnight to remove all residual water. The yield was 98%.

### Ionic liquid analysis

#### NMR

Analyses by using ^1^H and ^13^C NMR were carried out on a Bruker DRX 400 MHz instrument with dimethyl sulfoxide (DMSO) as solvent. In all cases, the chemical shifts were calibrated from the solvent peak [44] (DMSO ^1^H 2.54, ^13^C 40.45).

#### Karl-Fischer titration

The water content of the ILs was determined using a Metrohm 756 KF coulometer with diaphragm-free titration cell and Hydranal Coulomat E reagent. After exposure to high vacuum at 65°C overnight, approximately 100 μl of the sample was withdrawn into a dry syringe which was then weighed. Subsequently, the sample was injected into the Karl-Fischer reactor and re-weighed to determine the amount of sample injected.

### Pretreatment and enzymatic hydrolysis

#### Cellulosic substrates

Seven cellulosic and hemicellulosic substrates ranging from highly crystalline to amorphous were selected in order to achieve a broad evaluation. Wood samples included Norway spruce (kindly provided by SEKAB, Örnsköldsvik, Sweden) and hybrid aspen [kindly provided by the Umeå Plant Science Centre (UPSC), Umeå, Sweden]. Crystalline polysaccharides included cotton (Selefatrade AB, Spånga, Sweden) and Sigmacell Cellulose Type 20 (Sigma-Aldrich). Amorphous polysaccharides included beech-wood xylan (Sigma-Aldrich), galactomannan (locust bean gum, Sigma-Aldrich) and regenerated amorphous cellulose (prepared from Sigmacell cellulose Type 20 according to Zhang *et al.*[36]).

The wood samples, spruce and aspen, were milled using an IKA A11 Basic Analytical Mill and sieved (Retsch Analytical AS 200, 500–100 μm sieves). All cellulosic, hemicellulosic and lignocellulosic samples were preparatively dried using a vacuum pump until a dry matter content of ~100% was reached (Mettler Toledo HG63) thus avoiding water upon the IL pretreatment.

#### Pretreatment

Cellulosic samples (50 mg) were dispersed in 950 mg of ionic liquid and were then incubated for 20 hours at 100°C. The samples were allowed to cool to room temperature followed by the addition of up to 1 g of methanol in order to give a smoother initial precipitation. After that, 10 g of ultra-pure water (Millipore) was added followed by vigorous mixing and centrifugation for 10 min at 14,500  *g* (Eppendorf centrifuge 5810R). The supernatant was decanted and the biomass was subsequently washed using 3 × 10 g ultra-pure water and 1 × 10 g 50 mM citrate buffer pH 5.2 in a similar manner as previously stated. As a negative control, non-pretreated biomass was used. As a positive control, the milled and sieved aspen wood was pretreated at a combined severity of 2.2 using 1% (w/w) sulfuric acid as catalyst.

#### Enzymatic hydrolysis

The total weight of the reaction mixture was 1,670 mg and it contained 50 mg of the cellulosic/hemicellulosic sample, 50 mg enzyme mixture (a 1:1 ratio of a mixture of Celluclast 1.5 L and Novozyme 188, both of which were obtained from Sigma-Aldrich) and 50 mM citrate buffer (pH 5.2). The enzymatic hydrolysis was performed at 45°C in an orbital shaker set at 170 rpm (Ecotron incubator shaker, Infors). The samples (10 μl) were collected before enzyme addition (0 h), and after 2 h, 48 h and 72 h of enzymatic hydrolysis.

### Product analysis

#### Glucose concentration estimation

During the hydrolysis (samples taken after 0, 2, 48, and 72 h, respectively) the concentration of glucose was estimated using a glucometer (Accu-Chek Aviva, Roche Diagnostics GmBH). The glucose concentrations after 2 h were used for calculation of the GPR (the glucose production rate), which is determined before the hydrolysis reaction levels off.

#### Analysis of monosaccharide yields

The concentration of separate monosaccharides (arabinose, galactose, glucose, xylose, and mannose) was determined after 72 h of enzymatic hydrolysis using high-performance anion-exchange chromatography (HPAEC). A Dionex ICS-5000 equipped with an electrochemical detector, a CarboPac PA20 (3 × 30 mm) guard column and a CarboPac PA20 (3 × 150 mm) separation column (all from Dionex) were used to perform the analysis. Prior to analysis all samples were diluted using ultra-pure water and filtered through a 0.2 μm nylon membrane (Millipore). The concentrations determined with HPAEC were used for calculation of the monosaccharide yields, as the hydrolysis reactions had leveled off after 72 h.

#### Determination of structural carbohydrates and lignin in the wood samples

The extractives were determined according to Lestander *et al.*[45] with the exception that the extraction was performed in 15 cycles instead of during 1 h. After the extraction, the contents of carbohydrates (arabinan, galactan, glucan, mannan and xylan) and lignin (acid soluble and acid-insoluble lignin) were determined according to NREL/TP-510-42618 [46] with the exception that the concentration of the monosaccharides was determined using HPAEC (according to the procedure described in Section Analysis of monosaccharide yields).

## Abbreviations

HPAEC: High-performance anion-exchange chromatography; ILs: Ionic liquids; NMR: Nuclear magnetic resonance.

## Competing interests

The authors declare that they have no competing interests.

## Authors’ contributions

SW, JG and LJJ conceived and designed the study. The experimental work was carried out by JG, SW and MN under the supervision of LJJ and JPM. JG and SW contributed equally to this work and should therefore be considered as co-first-authors. All authors read and approved the final manuscript

## Authors’ information

JG is a doctoral student with focus on utilization of ionic liquids for conversion of lignocellulosic materials. SW is a postdoctoral researcher with interest in enzyme chemistry and technology. MN is a doctoral student with interest in the area of pretreatment and enzymatic saccharification of lignocellulose. LJJ is professor at Umeå University, and is working on biotechnology for biorefining of lignocellulosic feedstocks. He is also leader of the Biochemical Platform of the Bio4Energy research initiative (http://www.bio4energy.se). JPM is professor at Umeå University and Åbo Akademi University, and is working on ionic-liquid technologies and chemical catalysis for the biorefining of lignocellulose, e.g. within the Bio4Energy program.

## References

[B1] Perlack RD Wright LL Turhollow AF Graham RL Stokes BJ Erbach DC Biomass as a feedstock for a bioenergy and bioproducts industry: the technical feasibility of a billion-ton annual supply 2005 Springfield, VA: U.S. Department of Commerce, National Technical Information Service

[B2] Lichtenthaler FW Peters S Carbohydrates as green raw materials for the chemical industry C R Chim 2004 7 65 90 10.1016/j.crci.2004.02.002

[B3] Mosier N Wyman C Dale B Elander R Lee YY Holtzapple M Ladisch M Features of promising technologies for pretreatment of lignocellulosic biomass Bioresour Technol 2005 96 673 686 10.1016/j.biortech.2004.06.025 15588770

[B4] Chandra RP Bura R Mabee WE Berlin A Pan X Saddler JN Substrate pretreatment: the key to effective enzymatic hydrolysis of lignocellulosics? Adv Biochem Eng Biotechnol 2007 108 67 93 17530205 10.1007/10_2007_064

[B5] Zhang YHP Lynd LR Toward an aggregated understanding of enzymatic hydrolysis of cellulose: noncomplexed cellulase systems Biotech Bioeng 2004 88 797 824 10.1002/bit.20282 15538721

[B6] Biermann O Hadicke E Koltzenburg S Muller-Plathe F Hydrophilicity and lipophilicity of cellulose crystal surfaces Angew Chem Int Edit 2001 40 3822 3825 10.1002/1521-3773(20011015)40:20<3822::AID-ANIE3822>3.0.CO;2-V 11668544

[B7] Yamane C Aoyagi T Ago M Sato K Okajima K Takahashi T Two different surface properties of regenerated cellulose due to structural anisotropy Polym J 2006 38 819 826 10.1295/polymj.PJ2005187

[B8] Lindman B Karlström G Stigsson L On the mechanism of dissolution of cellulose J Mol Liq 2010 156 76 81 10.1016/j.molliq.2010.04.016

[B9] Liebert TF Heinze TJ Edgar KJ Cellulose solvents: for analysis, shaping and chemical modification 2009 Washington: American Chemical Society

[B10] Hermanutz F Gaehr F Uerdingen E Meister F Kosan B New developments in dissolving and processing of cellulose in ionic liquids Macromol Symp 2008 262 23 27 10.1002/masy.200850203

[B11] Heinze T Dicke R Koschella A Kull AH Klohr EA Koch W Effective preparation of cellulose derivatives in a new simple cellulose solvent Macromol Chem Phys 2000 201 627 631 10.1002/(SICI)1521-3935(20000301)201:6<627::AID-MACP627>3.0.CO;2-Y

[B12] Yan LF Chen J Bangal PR Dissolving cellulose in a NaOH/thiourea aqueous solution: a topochemical investigation Macromol Biosci 2007 7 1139 1148 10.1002/mabi.200700072 17683109

[B13] Yan LF Gao ZJ Dissolving of cellulose in PEG/NaOH aqueous solution Cellulose 2008 15 789 796 10.1007/s10570-008-9233-5

[B14] Liu SL Zhang LN Effects of polymer concentration and coagulation temperature on the properties of regenerated cellulose films prepared from LiOH/urea solution Cellulose 2009 16 189 198 10.1007/s10570-008-9268-7

[B15] Swatloski RP Spear SK Holbrey JD Rogers RD Dissolution of cellose with ionic liquids J Am Chem Soc 2002 124 4974 4975 10.1021/ja025790m 11982358

[B16] Tan SSY MacFarlane DR Ionic liquids in biomass processing Ionic Liquids 2009 290 311 339 10.1007/128_2008_35 21107802

[B17] Pinkert A Marsh KN Pang SS Staiger MP Ionic liquids and their interaction with cellulose Chem Rev 2009 109 6712 6728 10.1021/cr9001947 19757807

[B18] Brandt A Ray MJ To TQ Leak DJ Murphy RJ Welton T Ionic liquid pretreatment of lignocellulosic biomass with ionic liquid-water mixtures Green Chem 2011 13 2489 2499 10.1039/c1gc15374a

[B19] Sun N Rodriguez H Rahman M Rogers RD Where are ionic liquid strategies most suited in the pursuit of chemicals and energy from lignocellulosic biomass? Chem Commun 2011 47 1405 1421 10.1039/c0cc03990j 21170465

[B20] Zhao H Baker GA Song ZY Olubajo O Crittle T Peters D Designing enzyme-compatible ionic liquids that can dissolve carbohydrates Green Chem 2008 10 696 705 10.1039/b801489b

[B21] Turner MB Spear SK Huddleston JG Holbrey JD Rogers RD Ionic liquid salt-induced inactivation and unfolding of cellulase from Trichoderma reesei Green Chem 2003 5 443 447 10.1039/b302570e

[B22] Engel P Mladenov R Wulfhorst H Jager G Spiess AC Point by point analysis: how ionic liquid affects the enzymatic hydrolysis of native and modified cellulose Green Chem 2010 12 1959 1966 10.1039/c0gc00135j

[B23] Brandt A Hallett JP Leak DJ Murphy RJ Welton T The effect of the ionic liquid anion in the pretreatment of pine wood chips Green Chem 2010 12 672 679 10.1039/b918787a

[B24] Doherty TV Mora-Pale M Foley SE Linhardt RJ Dordick JS Ionic liquid solvent properties as predictors of lignocellulose pretreatment efficacy Green Chem 2010 12 1967 1975 10.1039/c0gc00206b

[B25] Gräsvik J Eliasson B Mikkola JP Halogen-free ionic liquids and their utilization as cellulose solvents J Mol Struct 2012 1028 156 163

[B26] Sun N Rahman M Qin Y Maxim ML Rodriguez H Rogers RD Complete dissolution and partial delignification of wood in the ionic liquid 1-ethyl-3-methylimidazolium acetate Green Chem 2009 11 646 655 10.1039/b822702k

[B27] Fort DA Remsing RC Swatloski RP Moyna P Moyna G Rogers RD Can ionic liquids dissolve wood? processing and analysis of lignocellulosic materials with 1-n-butyl-3-methylimidazolium chloride Green Chem 2007 9 63 69 10.1039/b607614a

[B28] Padmanabhan S Kim M Blanch HW Prausnitz JM Solubility and rate of dissolution for Miscanthus in hydrophilic ionic liquids Fluid Phase Equilibr 2011 309 89 96 10.1016/j.fluid.2011.06.034

[B29] Fukaya Y Hayashi K Wada M Ohno H Cellulose dissolution with polar ionic liquids under mild conditions: required factors for anions Green Chem 2008 10 44 46 10.1039/b713289a

[B30] Brandt A Gräsvik J Hallett JP Welton T Deconstruction of lignocellulosic biomass with ionic liquids Green Chem 2013 15 550 583 10.1039/c2gc36364j

[B31] Young RA Rowell RM Cellulose: structure, modification and hydrolysis 1986 New York: John Wiley and Sons

[B32] Fengel D Wegner G Wood: chemistry, ultrastructure, reactions 1989 New York: Walter de Gruyter

[B33] Krässig HA Cellulose: structure, accessibility and reactivity: polymer monographs vol. 11 1996 Amsterdam: Gordon and Breach Science Publishers

[B34] Van de Vyver S Thomas J Geboers J Keyzer S Smet M Dehaen W Jacobs PA Sels BF Catalytic production of levulinic acid from cellulose and other biomass-derived carbohydrates with sulfonated hyperbranched poly(arylene oxindole)s Energy Environ Sci 2011 4 3601 3610

[B35] Zhang Y-HP Lynd LR Determination of the number-average degree of polymerization of cellodextrins and cellulose with application to enzymatic hydrolysis Biomacromolecules 2005 6 1510 1515 10.1021/bm049235j 15877372

[B36] Zhang YHP Cui JB Lynd LR Kuang LR A transition from cellulose swelling to cellulose dissolution by o-phosphoric acid: evidence from enzymatic hydrolysis and supramolecular structure Biomacromolecules 2006 7 644 648 10.1021/bm050799c 16471942

[B37] Wang H Gurau G Rogers RD Ionic liquid processing of cellulose Chem Soc Rev 2012 41 1519 1537 10.1039/c2cs15311d 22266483

[B38] Kilpeläinen I Xie H King A Granstrom M Heikkinen S Argyropoulos DS Dissolution of wood in ionic liquids J Agr Food Chem 2007 55 9142 9148 10.1021/jf071692e 17907779

[B39] Zavrel M Bross D Funke M Buchs J Spiess AC High-throughput screening for ionic liquids dissolving (ligno-)cellulose Bioresour Technol 2009 100 2580 2587 10.1016/j.biortech.2008.11.052 19157872

[B40] Brandt A Ionic liquid pretreatment of lignocellulosic biomass 2012 London: Imperial College London

[B41] Troshenkova S Sashina E Novoselov N Arndt K Jankowsky S Structure of ionic liquids on the basis of imidazole and their mixtures with water Russ J Gen Chem 2010 80 106 111 10.1134/S1070363210010135

[B42] Saddler JN Bioconversion of forest and agricultural plant residues 1993 Wallingford: CAB International

[B43] Arora R Manisseri C Li CL Ong MD Scheller HV Vogel K Simmons BA Singh S Monitoring and analyzing process streams towards understanding ionic liquid pretreatment of switchgrass (Panicum virgatum L.) Bioenergy Res 2010 3 134 145 10.1007/s12155-010-9087-1

[B44] Gottlieb HE Kotlyar V Nudelman A NMR chemical shifts of common laboratory solvents as trace impurities J Org Chem 1997 62 7512 7515 10.1021/jo971176v 11671879

[B45] Lestander TA Geladi P Larsson SH Thyrel M Near infrared image analysis for online identification and separation of wood chips with elevated levels of extractives J Near Infrared Spec 2012 20 591 599 10.1255/jnirs.992

[B46] Sluiter A Hames B Ruiz R Scarlata C Sluiter J Templeton D Crocker D Determination of structural carbohydrates and lignin in biomass. Technical Report NREL/TP-510-42618 2011 Golden, CO: National Renewable Energy Laboratory

